# Calcium transport in bovine rumen epithelium as affected by luminal Ca concentrations and Ca sources

**DOI:** 10.14814/phy2.12615

**Published:** 2015-11-12

**Authors:** Bernd Schröder, Mirja R Wilkens, Gundula E Ricken, Sabine Leonhard-Marek, David R Fraser, Gerhard Breves

**Affiliations:** 1 Department of Physiology, University of Veterinary Medicine, FoundationHannover, Germany; 2 Faculty of Veterinary Science, University of SydneySydney, Australia

**Keywords:** Ca absorption, Ca sources, rumen wall, Ussing chamber

## Abstract

The quantitative role of different segments of the gastrointestinal tract for Ca absorption, the respective mechanisms, and their regulation are not fully identified for ruminants, that is, cattle. In different in vitro experiments the forestomach wall has been demonstrated to be a major site for active Ca absorption in sheep and goats. In order to further clarify the role of the bovine rumen for Ca transport with special attention to luminal Ca concentrations, its ionic form, and pH, electrophysiological and unidirectional flux rate measurements were performed with isolated bovine rumen epithelial tissues. For Ca flux studies (*J*_ms_, *J*_sm_) in vitro Ussing chamber technique was applied. Standard RT-PCR method was used to characterize TRPV6 and PMCA1 as potential contributors to transepithelial active Ca transport. At Ca concentrations of 1.2 mmol L^−1^ on both sides of the tissues, *J*_ms_ were higher than *J*_sm_ resulting under some conditions in significant Ca net flux rates (*J*_net_), indicating the presence of active Ca transport. In the absence of an electrical gradient, *J*_net_ could significantly be stimulated in the presence of luminal short-chain fatty acids (SCFAs). Increasing the luminal Ca concentrations up to 11.2 mmol L^−1^ resulted in significant increases in *J*_ms_ without influencing *J*_sm_. Providing Ca in its form as respective chloride, formate, or propionate salts there was no significant effect on *J*_ms_. No transcripts specific for Ca channel TRPV6 could be demonstrated. Our results indicate different mechanisms for Ca absorption in bovine rumen as compared with those usually described for the small intestines.

## Introduction

It is well accepted that there is significant calcium (Ca) absorption across the rumen wall of sheep and goats although the nature of the underlying mechanisms are not yet known (Wilkens et al. 2011, 2012). The importance of ruminal Ca transport in vivo seems to depend on the level of Ca intake. It could be demonstrated that the Ca net absorption before the duodenum rises with increasing levels of Ca intake in sheep and cattle (Khorasani et al. 1997; Schröder and Breves 2006).

In order to prevent or treat hypocalcemia in dairy cows, it is common practice to supply four doses of approximately 50 g of Ca in the form of a pharmaceutical bolus or gel 12 h before expected calving, immediately after calving, 12 h after calving, and 24 h thereafter (Oetzel 1996; Pehrson et al. 1998). This oral administration of Ca salts can raise ruminal Ca concentrations up to 10 mmol L^−1^. Ca chloride is probably the most commonly used compound, but as it can affect the integrity of gastrointestinal epithelia, other anions like propionate or formate have also been tested in vivo (Goff and Horst 1993; McIntyre and Weston 2002). After oral application, Ca chloride seems to increase plasma Ca more rapidly than Ca propionate (Goff and Horst 1994), but a direct effect of the Ca source on its gastrointestinal absorption has not been investigated in cattle so far.

It was the aim of this study to examine whether the Ca source, its concentration, and luminal pH might have direct effects on Ca transport across the bovine rumen wall. As most of the former studies on ruminal Ca transport were performed with tissues from sheep and goats, basic functional and structural investigations were carried out additionally in order to clarify whether data on ruminal Ca absorption obtained from small ruminant species also apply in cattle.

## Materials and Methods

### Tissue sampling

Tissues were obtained from cattle immediately after slaughtered at the local abattoir. For Ussing chamber experiments, a piece of about 20 × 25 cm was taken from the ventral rumen wall within 20 min after stunning and exsanguination and rinsed with the respective mucosal buffer solution (depending on the experiment buffer B, D, or E as shown in Table1) at 40°C. The mucosa was stripped immediately from the underlying muscle and serosal layers and kept in buffer solution at 38°C oxygenated with carbogen (95% O_2_/5% CO_2_) until it was mounted into the Ussing chambers 15 min later.

**Table 1:** Composition of the buffer solutions

Substance	Buffer A	Buffer B	Buffer C^1,2,3^	Buffer D	Buffer E
Na^+^	143.2	143.2	143.2	140.0	141.8
Cl^−^	67.2	67.6	67.6–72.68^**1**^ 65.2^**2,3**^	120.0	24.9–44.9
Ca^2+^	1.2	1.2	1.2–3.74^**1,2,3**^	1.2	1.2–11.2
K^+^	5.0	5.0	5.0	5.0	5.0
Mg^2+^	1.2	1.2	1.2	1.2	1.2
HCO_3_^−^	21.0	21.0	2.0	21.0	2.0
HPO_4_^2−^	1.4	1.2	0.5	2.4	0.3
H_2_PO_4_^−^	1.4	1.6	2.3	0.6	2.7
Glucose	5.0	–	–	10.0	5.0
Formate	–	–	1.2–3.74^**2**^	–	–
Acetate	–	36.0	36.0	–	78.0
Propionate	–	15.0	16.2–18.74^**3**^	–	30.0
Butyrate	–	9.0	9.0	–	12.0
Gluconate	61.0	1.2	20.9	–	–
PH	7.40	7.44	6.55	7.40	6.52

Concentrations are given in mmol L^−1^. For further information see Materials and Methods section. The superscripts refer to different buffer solutions which are explained in more detail in the “Materials and Methods” section.

For isolation of RNA, further samples from the ventral rumen wall and the duodenum as a positive control were rinsed with ice-cold physiological saline. Ruminal mucosa was stripped manually from the underlying layers. The samples from duodenum were placed on ice, where the mucosa was separated from the submucosal layers using glass slides. The prepared samples were instantly frozen in liquid nitrogen and stored at −80°C.

### Ussing chamber technique

In vitro studies on Ca flux rates across ruminal epithelia were carried out using the Ussing chamber technique as described elsewhere (Schröder et al. 1997). Shortly, tissues were mounted between the two halves of standard Ussing chambers with an exposed area of 2 cm^2^ and protected from potential edge damage by rings of silicon rubber. Thereby, the Ussing chambers were separated into a serosal and a mucosal compartment each connected to circulation reservoirs filled with respective buffer solutions which were maintained at 39°C by water jackets and continuously stirred and oxygenated with carbogen by a gas lift system.

A computer-controlled voltage clamp device (Mussler Scientific Instruments, Aachen, Germany) was used to measure transepithelial potential differences (PD_t_), tissue conductances (*G*_t_), and short-circuit currents (*I*_sc_). Unless otherwise noted, the experiments were carried out under short-circuit current conditions. This means that the transepithelial potential difference originally generated by active net electrogenic transport processes was clamped to 0 mV.

### Determination of Ca flux rates

Ca flux rates were determined with ^45^Ca. For this purpose, 185 kBq (5 *μ*Ci) were added to each chamber as ^45^CaCl_2_ (original specific activity >370 GBq g^−1^; PerkinElmer Life Sciences, Rodgau-Jürgensheim, Germany). After an equilibration period of 25 min, eight samples of 500 *μ*L were taken at the intervals of 30 min. The sample volume was immediately replaced by an aliquot of the respective buffer solution. Radioactivity of the samples was measured using a conventional liquid scintillation counter (Tricarb Packard, Dreieich, Germany). Unidirectional flux rates from the mucosal to the serosal side (*J*_ms_) and from the serosal to the mucosal side (*J*_sm_) were calculated from the rate of tracer appearance using standard equations (Schultz and Zalusky 1964). To determine net flux rates (*J*_net_), *J*_sm_ were subtracted from respective *J*_ms_. Since *J*_sm_ remained constant irrespective of the experimental conditions, only *J*_ms_ were measured in the second set of experiments studying the effects of different Ca salts on Ca transport.

### Buffer solutions

The compositions of the different buffer solutions are presented in Table1. All solutions had an osmolality of 300 mOsmol kg^−1^. The given pH values were determined in solutions oxygenated with carbogen at a temperature of 39°C. All chemicals were of analytical grade and were obtained from Merck (Darmstadt, Germany) or Sigma-Aldrich Chemicals (St. Louis, USA).

### Experimental design of Ussing chamber studies

#### Unidirectional Ca flux rates in the absence and presence of luminal SCFA

In order to investigate potential effects of short-chain fatty acids (SCFAs) in physiological proportions on Ca flux rates, either a SCFA-free (Table1, buffer A) or a SCFA buffer with 36.0, 15.0, and 9.0 mmol L^−1^ Na acetate, Na propionate, and Na butyrate (Table1, buffer B), respectively, was used as the luminal buffer solution. The same buffer was present on the serosal side in all chambers (Table1, buffer A).

#### Effect of different anions under physiological conditions

To elucidate alterations of Ca flux rates by different anions under conditions similar to the in vivo situation, the serosal compartments of the Ussing chambers were filled with modified Krebs–Henseleit solution (Table1, buffer A). The mucosal buffer contained SCFAs and the pH was adjusted to 6.55 in order to simulate a physiological intra-ruminal pH range. Ca sources in the mucosal buffer were either Ca chloride, Ca formate, or Ca propionate (Table1, buffer C^1,2,3^). After a control period of 30 min, the Ca concentration in the mucosal buffer was raised from 1.2 to 3.74 mmol L^−1^ by adding the respective Ca salt.

#### Simulation of a pharmaceutical application of a Ca bolus

In this experiment, the serosal compartment contained a Krebs–Henseleit buffer (Table1, buffer D), whereas the mucosal side of the epithelium was incubated in a buffer solution, which contained SCFAs in physiological proportions (Table1, buffer E). The luminal pH was adjusted to 6.52; after a control period of 30 min, the Ca concentration in the mucosal buffer was raised from 1.2 to 11.2 mmol L^−1^ by addition of Ca chloride.

### Determination of ionized Ca

For the determination of ionized Ca (Ca^2+^), respective buffer solutions (Table1, buffer C) at 37°C were oxygenated with carbogen in the lift system of the Ussing chamber for 30 min. This procedure should ensure to adjust buffer Ca^2+^ under experimental Ussing chamber conditions. Samples taken from these solutions were transferred to a blood gas and electrolyte analyzer (Rapidlab 248, Chiron Diagnostrics GmbH, Fernwald, Germany).

### Isolation of RNA and reverse transcription

The kit for isolation of RNA contained special spin columns to eliminate gDNA (Qiagen, Hilden, Germany). Primer pairs were always designed to amplify products that span at least one intron, therefore, “NTC” *was* carried out using water instead of a template. Total RNA was isolated using the RNeasy Mini-Kit (Qiagen, Hilden, Germany) according to the manufacturer’s protocol. Concentration and quality of RNA were determined by UV absorbance and 200 ng were reverse transcribed using random hexamer primers and TaqMan-Reverse Transcription Reagents (Applied Biosystems, Darmstadt, Germany).

### RT-PCR

Products specific for TRPV6 (transient receptor potential vanilloid type 6) and PMCA1 (plasma membrane Ca-ATPase type 1) were followed up with RT-PCR. Amplification of the specific products was carried out using standard PCR conditions (1.5 mmol L^−1^ MgCl_2_, 0.2 mmol L^−1^ dNTPs, 0.3 μmol L^−1^ sense primer, 0.3 μmol L^−1^ antisense primer, 0.02 U *μ*L^−1^ *Taq* polymerase) and protocols (5 min at 94°C; 30 cycles of 30 sec at 94°C, 30 sec at 55°C and 90 sec at 72°C; 5 min at 72°C). Primer pairs were always designed to amplify products that span at least one intron, therefore, “NTC” *was* carried out using water instead of a template. Oligonucleotides for amplification of the product specific for TRPV6 (sense primer: 5′-GGATCTGTGGGCACAAGTTT-3′; antisense primer: 5′-CGGGAGGTACTTCGAGACAC-3′) were designed based on available sequence information (XM_866519). Oligonucleotides for amplification of PMCA1 (sense primer: 5′-TAGGCACTTTTGTGGTACAG-3′; antisense primer: 5′-GCTCTGAATCTTCTATCCTA-3′) and GAPDH, which was used as internal control (sense primer: 5′-TGTTCCAGTATGATTCCACCC-3′; antisense primer: 5′-TCCACCACCCTGTTGCTGTA-3′) were synthesized by Invitrogen GmbH (Karlsruhe, Germany) according to the published data (Bian et al. 2000; Tsai et al. 2001).

The PCR product specific for TRPV6 was purified using the Jetsorb Gel Extraction Kit (Genomed GmbH, Bad Oenhausen, Germany), while the PCR products specific for PMCA1 and GAPDH were prepared using the MinElute PCR Purification Kit (Qiagen, Hilden, Germany). All products were sequenced by GATC Biotech AG (Konstanz, Germany). The sequence confirmation was verified by NCBI Blast.

### Statistical analyses

After confirmation of Gaussian distribution according to Kolmogorov–Smirnov (if *N* = 6), data were compared using one-way analysis of variance (ANOVA), two-way ANOVA (Ca source, Ca concentration, and interaction), or paired *t*-test (effect of the Ca bolus), respectively. With *N* = 4 (chapter “effect of different anions”) nonparametric one-way ANOVA on the basis of Kruskal–Wallis test was performed (GraphPad Prism Version 4.03, San Diego). Values are given as means ± SEM. *N* indicates the number of animals.

## Results

### Ussing chamber experiments

#### Basic electrophysiological data

There were slight differences in the basal electrophysiological properties of rumen tissues from the slaughtered cattle. During the measurement period mean transepithelial conductances varied between 9.93 and 10.63 mS cm^−2^, but showed no changes during the experiments. Replicate tissue samples showed little variability in conductances. Short-circuit currents ranged from 0.66 to 0.80 *μ*eq cm^−2^ h^−1^ and showed only small variations during the experiments. The addition of ouabain (0.1 mmol L^−1^) to the serosal side at the end of the experiments reduced the *I*_sc_ by more than 75% thus demonstrating tissue viability under the experimental conditions.

#### Unidirectional Ca flux rates in the absence and presence of luminal SCFA

Ca net flux rates (*J*_net_) of Ca flux rates of tissues incubated in control buffer without SCFA amounted to 3.16 ± 0.90 nmol cm^−2^ h^−1^ (*N* = 6). The presence of SCFA in the mucosal buffer increased *J*_ms_ by about 60% without affecting *J*_sm_ (Table2) resulting in *J*_net_ of 13.75 ± 1.80 nmol cm^−2^ h^−1^.

**Table 2:** Ca flux rates at different Ca concentrations and with different Ca salts on the mucosal side

SCFA muc	Buffer muc/ser	pH muc	Ca salt	Ca mmol L^−1^	*J*_ms_	*J*_sm_	Ca mmol L^−1^	*J*_ms_ nmol cm^−2^ h^−1^	*N*
					nmol cm^−2^ h^−1^				
−	A/A	7.40	Ca chloride	1.2	15.98 ± 1.76	12.82 ± 1.62			6
+	B/A	7.44	Ca chloride	1.2	25.08 ± 3.10	11.33 ± 2,24			6
+	C/A	6.55	Ca chloride	1.2	16.4 ± 8.0		3.74	73.7 ± 25.1	4
+	C/A	6.55	Ca formate	1.2	16.4 ± 9.8		3.74	73.0 ± 33.6	4
+	C/A	6.55	Ca propionate	1.2	16.0 ± 6.0		3.74	71.8 ± 16.4	4
+	E/D	6.52	Ca chloride	1.2	10.66 ± 1.06	9.62 ± 0.62	11.2	111.90 ± 13.37	6

Data are mean ± SEM. *N* = number of animals. For the composition of buffer solutions see Table1.

#### Effect of different anions under physiological conditions

Providing 1.2 mmol L^−1^ Ca in its form as chloride, formate, or propionate salt in the presence of 65 mmol L^−1^ Cl, 36 mmol L^−1^ acetate, 15 mmol L^−1^ propionate, and 9 mmol L^−1^ butyrate resulted in comparable *J*_ms_ when added in a concentration of 1.2 mmol L^−1^ (Table2). Higher amount of these Ca salts (3.74 mmol L^−1^) resulted in higher *J*_ms_ with no significant differences between the three salts (Table2). The electrophysiological parameters remained unaffected (data not shown).

#### Ionized calcium

The fractions of Ca^2+^ measured in buffer solutions C^1,2,3^ in the presence of 65 mmol L^−1^ Cl^−^, 60 mmol L^−1^ SCFAs, 20 mmol L^−1^ gluconate ranged between 55% and 59% of total Ca. A further addition of the different anions, namely chloride, propionate, and formate in concentrations between 1.20 and 3.74 mmol L^−1^ thus had no significant effect on the proportion of Ca^2+^ ions.

#### Simulation of a Ca bolus application

Under the basal conditions of this experiment (no short-circuit current but open-circuit condition [indicating in vivo conditions], SCFA gradient: 120 mmol L^−1^ mucosal, no SCFAs at the serosal side, and pH gradient: 6.52 mucosal, 7.4 serosal), *J*_ms_ and *J*_sm_ Ca flux rates were in the same order of magnitude (Table2). Raising the mucosal Ca concentration from 1.2 to 11.2 mmol L^−1^ increased the Ca flux rates from mucosal to serosal from 10.66 ± 1.06 to 111.90 ± 13.37 nmol cm^−2^ h^−1^ (*N* = 6), without affecting *J*_sm_. The transepithelial conductance remained stable between 5.16 ± 0.56 mS cm^−2^ at the beginning and 4.96 ± 0.55 mS cm^−2^ at the end of this experiment, that is, after addition of the Ca bolus.

### RT-PCR

**Figure 1:**
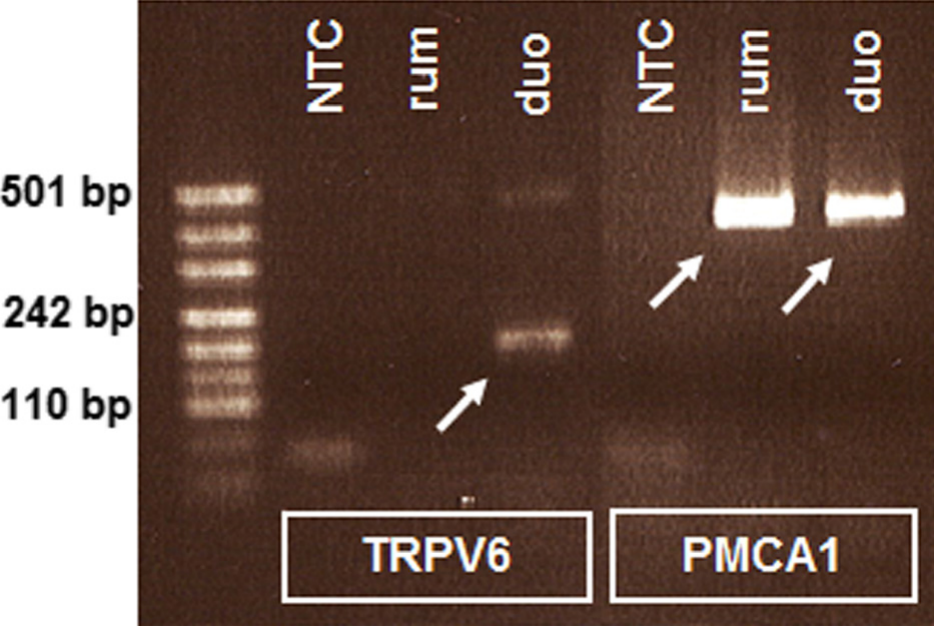
RT-PCR for the detection of products specific for TRPV6 and PMCA1. NTC, no template control; rum, rumen; duo, duodenum.

By means of RT-PCR, transcripts specific for TRPV6 could be demonstrated in the bovine duodenum, but were not found in ruminal tissues. However, transcripts specific for PMCA1 could be detected in both, the duodenum and the rumen (Fig.1). Amplification of GAPDH was carried out to confirm the integrity of the cDNA (data not shown).

## Discussion

### Ca transport at low luminal Ca concentration

In the presence of 1.2 mmol L^−1^ Ca on both sides of the tissues, that is, in the absence of a transepithelial gradient for Ca, significant *J*_net_ indicate the presence of active Ca transport across the bovine rumen wall and is thus in accordance with respective data from small ruminants (Höller et al. 1988; Uppal et al. 2003). The stimulatory effects of SCFAs on active Ca transport have already been demonstrated in similar studies with ovine rumen tissues. In these experiments it could be shown that the individual SCFA differed with regard to their stimulatory effect, that is, butyrate resulted in higher *J*_net_ than respective concentrations of acetate and propionate and the highest values were detected in the presence of the same SCFA mixture as in the present study (Schröder et al. 1999). The dependency of active ruminal Ca transport from SCFAs suggests the presence of a Ca^2+^/H^+^ exchange system at the apical membrane similarly as it has already been discussed for the rat hindgut (Lutz and Scharrer 1991).

### Ca absorption at high luminal Ca concentration

Increasing the Ca concentrations in the luminal solution to 3.74 and 11.2 mmol L^−1^ induced prompt increases in net Ca flux rates across bovine rumen epithelium. This was due to several-fold increases in *J*_ms_, whereas the secretory component (*J*_sm_) was unaffected by approximately 10-fold changes in Ca concentration in the luminal solution. In the intact animal this observation would mean that an increase in ruminal Ca concentration by the application of Ca salts (as gel or bolus) would immediately increase ruminal Ca absorption, which is consistent with the rapid increase in serum Ca reported in the past (Goff and Horst 1994).

The supply of high concentrations of ionized Ca to the mucosal side of the Ussing preparations allowed more Ca^2+^ to be presented to the absorbing cells and hence an increase in active or passive transport could be demonstrated even in the absence of mucosal motility and blood circulation.

### Stimulating effect of anions

An increase in Ca concentration from 1.2 to 3.74 mmol L^−1^ (3.1-fold) increased *J*_ms_ 4.5-fold, whereas a further increase in Ca concentration from 1.2 to 11.2 mmol L^−1^ (9.3-fold) increased *J*_ms_ 10.5-fold. It is therefore possible that the relatively higher increase in flux rates compared to the increase in luminal Ca concentration might point to an additional effect of anions as shown earlier for Ca absorption across the rumen epithelium of sheep (Schröder et al. 1999; Uppal et al. 2003; Leonhard-Marek et al. 2007). However, there were no differences in Ca transport when different anions were supplied. This suggests that changes in the flux rates are either independent of the anionic environment or that the changes in anion concentrations were too small in relation to the basal anion concentrations of the buffer solutions.

### Structural equivalents of Ca transport

It is generally accepted that active, transcellular Ca^2+^ transport across intestinal epithelia is at least a three-step process involving the passive entry of Ca^2+^ via the transient receptor potential vanilloid channel type 6 (TRPV6) (Peng et al. 1999; see Dimke et al. 2011; for review), the cytosolic transfer of Ca^2+^ via contribution of calbindin D_9K_ (CaBP_9K_) (Bronner 1987), and the extrusion of Ca^2+^ across the basolateral membrane via a Ca^2+^-ATPase (PMCA1) (van Abel et al. 2003; see Diaz de Barboza et al. 2015, for review).

The epithelial Ca^2+^ channel TRPV6 on the luminal cellular membrane was detected at both mRNA and protein level in the small intestine but only trace amounts were found in the rumen of sheep (Wilkens et al. 2009). In the present study, transcripts specific for TRPV6 could only be demonstrated in the duodenum, while there was no expression in the rumen. Against the background of these results as well as the expression patterns of TRPV6 in ovine ruminal epithelia, a significant contribution of TRPV6 to Ca transport across the forestomach appears questionable. The expression of RNA specific for CaBP_9K_ and the PMCA1 has already been demonstrated in duodenal tissue samples from dairy cattle (Yamagishi et al. 2006). CaBP_9K_ protein could be further shown in mucosal cells of ovine and caprine jejunum but not in rumen (Schröder et al. 2001; Wilkens et al. 2011, 2012). In the present study, we were able to confirm the expression of RNA specific for PMCA1 in bovine duodenum and we were able to detect expression of PMCA1 transcripts in the rumen of cattle. However, since the expression of PMCA1 is ubiquitous and knockout mutations lead to embryonic death (Okunade et al. 2004), this protein might have a housekeeping role rather than a significant function in Ca transport across the rumen wall. This speculation is supported by the observation that vanadate, the potential inhibitor of Ca^2+^-ATPase function, did not alter ruminal Ca transport in growing goats (Schröder et al. 1999).

Based on these findings, it is very likely that the Ca transport across rumen epithelia that has been demonstrated for various ruminant species is not mediated via the classical model described for intestinal epithelia. Recent studies with knockout mice have shown that active Ca absorption remains possible even in the absence of TRPV6 and CaBP_9K_. Using the everted gut sac assay, no differences in duodenal active Ca transport could be detected between wild-type, TRPV6 knockout, CaBP_9K_ knockout, and TRPV6/CaBP_9K_ double knockout mice fed a standard rodent chow diet (Benn et al. 2008). For the rumen epithelium, we could show recently the existence of a nonspecific cation conductance which might contribute to the absorption of Ca across the rumen wall (Leonhard-Marek 2002; Leonhard-Marek et al. 2005, 2010).

## Conclusion

Despite indication of active and passive Ca transport across bovine rumen, the respective epithelial mechanisms are not yet characterized. There seems to be no major role for TRPV6 in Ca absorption across the rumen of cattle. Alternatively, Ca transport might be performed through apical nonspecific cation channels and/or Ca^+^/H^+^-exchange or paracellular conductances. Irrespective of the underlying mechanism, ruminal Ca absorption can be increased immediately by an increase in ruminal Ca concentration at least between 1 and 11 mmol L^−1^ (corresponding to a bolus of 40 g Ca given into a rumen volume of 100 L). Apparently the ionic source has no significant effect on Ca transport at least in cattle. Most important for the increase in Ca absorption is the increase in the concentration of ionized Ca within the rumen by the given supplement, although ruminal motility and blood circulation in vivo may enhance calcium absorption across the rumen mucosa in a manner not seen in static mucosal preparations in vitro.
